# SIMPLE binds specifically to PI4P through SIMPLE-like domain and participates in protein trafficking in the trans-Golgi network and/or recycling endosomes

**DOI:** 10.1371/journal.pone.0199829

**Published:** 2018-06-28

**Authors:** Yasuhiro Moriwaki, Yuho Ohno, Tomohiro Ishii, Yuki Takamura, Yuko Kita, Kazuhiko Watabe, Kazunori Sango, Shoutaro Tsuji, Hidemi Misawa

**Affiliations:** 1 Division of Pharmacology, Faculty of Pharmacy, Keio University, Minato-ku, Tokyo, Japan; 2 Laboratory for Neurodegenerative Pathology, Tokyo Metropolitan Institute of Medical Science, Setagaya-ku, Tokyo, Japan; 3 Diabetic Neuropathy Project, Tokyo Metropolitan Institute of Medical Science, Setagaya-ku, Tokyo, Japan; 4 Molecular Diagnostics Project, Kanagawa Cancer Center Research Institute, Yokohama, Kanagawa, Japan; Institut Curie, FRANCE

## Abstract

Small integral membrane protein of the lysosome/late endosome (SIMPLE) is a 161-amino acid cellular protein that contains a characteristic C-terminal domain known as the SIMPLE-like domain (SLD), which is well conserved among species. Several studies have demonstrated that SIMPLE localizes to the trans-Golgi network (TGN), early endosomes, lysosomes, multivesicular bodies, aggresomes and the plasma membrane. However, the amino acid regions responsible for its subcellular localization have not yet been identified. The SLD resembles the FYVE domain, which binds phosphatidylinositol (3)-phosphate (PI3P) and determines the subcellular localization of FYVE domain-containing proteins. In the present study, we have found that SIMPLE binds specifically to PI4P through its SLD. SIMPLE co-localized with PI4P and Rab11, a marker for recycling endosomes (REs, organelles enriched in PI4P) in both the IMS32 mouse Schwann cell line and Hela cells. Sucrose density-gradient centrifugation revealed that SIMPLE co-fractionated with syntaxin-6 (a TGN marker) and Rab11. We have also found that SIMPLE knockdown impeded recycling of transferrin and of transferrin receptor. Our overall results indicate that SIMPLE may regulate protein trafficking physiologically by localizing to the TGN and/or REs by binding PI4P.

## Introduction

Vesicular trafficking, essential for delivering proteins and lipids to their correct destination, affects diverse signal transduction pathways [1–3]. Cells internalize extracellular cargo such as ligands, plasma membrane proteins and lipids through endocytosis (endocytic pathway). Then, within the endocytic pathway, these internalized molecules first enter early endosomes (EEs) and are either returned to the cell surface (recycling pathway) in recycling endosomes (REs), or are transported to late endosomes (LE) and lysosomes for degradation (degradation pathway). It is well known that intracellular organelles, such as EEs and REs, contain specific phosphoinositide species that are essential for the localization and function of their binding partner proteins [2, 4]. For example, phosphatidylinositol (3)-phosphate (PI3P) localizes specifically in EEs and defines the localization of PI3P-binding proteins containing a FYVE domain. FYVE domain-containing proteins regulate the transition of cargos between EEs and LEs [5, 6]. PI4P localizes specifically within the TGN and/or REs and defines the localization of EHD family proteins. EHD1 proteins regulate the recycling of PI4P-binding proteins and lipids from REs to the plasma membrane [7].

Small integral membrane protein of the lysosome/late endosome (SIMPLE), also known as lipopolysaccharide-induced TNF-α factor (LITAF) and p53-inducible gene-7 (PIG-7) is a 161-amino acid (aa) cellular protein that includes a characteristic C-terminal domain termed the SIMPLE-like domain (SLD) [8–10]. The SLD is rich in cysteines and resembles the RING domain, which is thought to mediate E3 ubiquitin ligase activity [11], as well as the FYVE domain, except that SLD is interrupted by a hydrophobic transmembrane (TM) domain [8]. SLD is found in a wide variety of species, including plants, insects, and mammals, and defines a new family of proteins with unknown function [8]. Ho *et al*. recently reported that the 114–139 aa hydrophobic helical region within the SLD is responsible for membrane tethering [12]. Moreover, they and others have revealed that SIMPLE is a monotopic zinc-binding protein, and that the function of the cysteine residues outside the 114–139 aa hydrophobic helical region is to mediate zinc binding for stabilization of the protein structure [12, 13]. However, given the evidence collected by Lee and colleagues that SIMPLE does not have E3 ubiquitin ligase activity [14], the exact role of the SLD remains to be elucidated.

Previous reports showed that SIMPLE localizes in EEs and regulates endosomal sorting complexes required for transport (ESCRT)-dependent multivesicular body (MVB) sorting of activated epidermal growth factor receptor [15]. On the other hand, another group reported that SIMPLE localizes in MVBs and is involved in the regulation of exosome production [16]. Aside from EEs and MVBs, SIMPLE has also been detected within the trans-Golgi network (TGN) [17, 18], lysosomes [8, 12, 13, 16, 19–21], aggresomes [22], and the plasma membrane [18], though the function of SIMPLE within organelles other than EEs and MVBs remains unclear.

In the present study, we show that SIMPLE binds PI4P through the SLD domain and localizes in TGN and/or REs, which are enriched in PI4P, in both IMS32 and Hela cells. Moreover, SIMPLE regulates protein trafficking, as demonstrated by a transferrin trafficking assay. Thus, SIMPLE could contribute to the protein recycling pathway.

## Materials and methods

### Purification of recombinant human SIMPLE and several truncation mutants

The cDNA encoding human SIMPLE was reverse transcribed using standard RT-PCR cloning procedures. Several SIMPLE truncation mutants were generated using PCR-based mutagenesis strategies and were confirmed by dsDNA sequencing. An anagram of SIMPLE (115–141) was synthesized by Geneart Gene Synthesis (Thermo Fisher Scientific, Inc., Rockford, IL, USA). The full-length, truncated, and anagram SIMPLE sequences were subcloned into the EcoRI and NdeI sites of a pMAL-c5x vector (New England BioLabs, Beverly, MA) in frame with the N-terminal MBP tag, and then used to transform competent *E*. *coli* BL21(DE2) containing pGKJE8 (TaKaRa Bio Inc., Shiga, Japan). Recombinant fusion proteins were purified from bacterial lysates using column chromatography with amylose resin, applying the method advocated by the supplier (New England BioLabs). The column buffer contained 20 mM Tris-HCl [pH 7.4], 200 mM NaCl, 1 mM sodium azide, 10 mM 2-mercaptoethanol and Complete Protease Inhibitors (Roche Diagnostics, Mannheim, Germany). The eluted fraction containing 10 mM maltose was dialyzed against Tris-buffered saline (TBS). The purified proteins were subjected to SDS-PAGE and stained with Coomassie Brilliant Blue (CBB).

### Antibody preparation

Mouse SIMPLE cDNA without the C-terminal hydrophobic TM region (mSIMPLEΔTM) was subcloned into the BamHI and XhoI sites of a pGEX6p-1 vector (GE Healthcare, Madison, WI, USA) in frame with an N-terminal glutathione-S-transferase (GST) tag and used to transform BL21(DE3)pLysS competent *E*. *coli* (Promega, Madison, WI, USA). GST-tagged mSIMPLEΔTM was then purified from bacterial lysate using glutathione Sepharose 4B (GE Healthcare) chromatography according to the manufacturer’s instructions.

Polyclonal antibody (pAb) against mouse SIMPLE was generated by immunizing a rabbit with GST-tagged mSIMPLEΔTM following standard methods. A monoclonal antibody (mAb) against human SIMPLE was generated through immunization with MBP-tagged human SIMPLE in combination with an Addavax adjuvant (Invivogen, San Diego, CA, USA) and hybridoma fusion, as described previously [38].

### Phospholipid binding using PIP strips

PIP strips (Echelon Biosciences Inc., Salt Lake City, UT) were blocked in 3% fatty acid-free bovine serum albumin (BSA, Sigma-Aldrich, St Louis, MO, USA) in TBST (TBS containing 0.05% Tween 20) for 1 h at room temperature. The membrane was then incubated for 18 h at 4°C with 5 nM MBP or MBP fusion proteins in TBST containing 3% fatty acid-free BSA, washed, and immunoblotted with anti-MBP mAb (1:4000 dilution; New England BioLabs). Immunopositive proteins were detected using ECL Plus Western Blotting Detection Reagent (GE Healthcare).

### Cell culture and transfection

IMS32 immortalized adult mouse Schwann cells (Gifted from Dr. Watabe, 33) and Hela cells (RIKEN Cell Bank, Tsukuba, Japan) were cultured at 37°C in Dulbecco’s modified Eagle’s medium (DMEM) supplemented with 5% fetal bovine serum (FBS), 100 U/mL of penicillin and 100 μg/mL of streptomycin. To knock down SIMPLE, cells were transfected with 25 nM siRNA using Lipofectamine 2000 (Thermo Fisher Scientific, Inc.) according to the manufacturer’s instructions, and then cultured for 48 h. siRNAs against murine SIMPLE (sense, 5'-CACAUACAGAUAAGGUCACAAGU-3', antisense, 5'-ACUUGUGACCUUAUCUGUAUGUG-3') and human SIMPLE (sense, 5'-GCAUGAAUCCUCCUUCGUAUU-3', antisense, 5'-UACGAAGGAGGAUUCAUGCCC-3'), and negative control siRNA (SIC-001) were purchased from Sigma-Aldrich. To express EGFP-human Rab11a and EGFP-human SIMPLE, pEGFP-C1 vectors encoding human Rab11a cDNA and human SIMPLE cDNA were transfected using Lipofectamine 2000 according to the manufacturer’s instructions.

### Western blotting and antibodies

Western blot analysis was performed using standard techniques described previously [39]. Briefly, cells were lysed in RIPA buffer (1% Triton X-100, 0.5% sodium deoxycholate, 0.1% SDS, 0.15M NaCl, 1 mM EDTA, 20 mM HEPES [pH 7.4]) supplemented with Complete Protease Inhibitors (Roche Diagnostics), after which equal amounts of protein (30 μg each) from the lysate were subjected to SDS-PAGE. The separated proteins were then transferred to polyvinylidene difluoride membranes (Immobilon-P; Merck Millipore, Billerica, MA, USA) and were probed with specific primary antibodies along with appropriate HRP-conjugated secondary antibodies (Bio-Rad Laboratories, Inc., Hercules, CA, USA). Immunopositive proteins were detected using ECL Plus Western Blotting Detection Reagent (GE Healthcare). As a loading control, membranes were probed using a mAb against β-actin (MAB1501, 1:1000 dilution; Merck Millipore). The antibodies used in this study were as follows: anti-Rab11 mAb (47, 1:500 dilution; BD Biosciences, Franklin Lakes, NJ, USA), anti-Syntaxin-6 mAb (1:500 dilution; Cell Signaling Technology, Inc., Danvers, MA, USA), anti-EEA1 pAb (1:500 dilution; Cell Signaling Technology, Inc.), anti-TSG101 mAb (4A10, 1:500 dilution; Genetex, San Antonio, TX, USA), anti-NEDD4 pAb (1:500 dilution; Cell Signaling Technology, Inc.), anti-TfnR mAb (H68.4, 1:1000 dilution; Thermo Fisher Scientific, Inc.). Anti-human SIMPLE mAb (1:1000 dilution) and rabbit anti-mouse SIMPLE pAb (1:500 dilution) were made in our laboratories.

### Immunocytochemistry

Immunofluorescence analyses of PI4P and Rab11 were performed as described previously, with minor modifications [25, 40]. To stain PI4P, cells grown on coverslips were first fixed for 15 min with 2% paraformaldehyde (PFA) in 0.1 M phosphate buffer (PB) at pH 7.4. They were then washed three times with phosphate buffered saline (PBS) containing 50 mM NH_4_Cl and permeabilized for 5 min with 20 μM digitonin in buffer A (20 mM PIPES [pH 6.8] containing 137 mM NaCl and 2.7 mM KCl) and blocked for 1 h with a blocking buffer (5% normal goat serum in PBS containing 50 mM NH_4_Cl). Primary antibodies (anti-PI4P mAb, 1:60 dilution, Echelon Biosciences Inc.; anti-mouse SIMPLE pAb, 1:100 dilution; anti-human SIMPLE mAb, 1:100 dilution) were applied for 1 h in the blocking buffer. After two washes in buffer A, secondary antibodies were added for 45 min in the blocking buffer. The cells were then incubated with 4',6'-diamidino-2-phenylindole dihydrochloride (DAPI) (Sigma-Aldrich) to visualize nuclei, washed again and post-fixed for 10 min with 2% PFA in 0.1 M PB. PFA was removed by washing three times with PBS containing 50 mM NH_4_Cl, after which the coverslips were mounted using Fluoremount-G (SouthernBiotech, Birmingham, AL, USA) and examined using an Olympus FV-1000 confocal system (Tokyo, Japan) equipped with a 60x objective lens (N.A. = 1.35).

To stain Rab11, cells were fixed for 10 min with 4% PFA in 0.1 M PBS, quenched with 50 mM NH_4_Cl for 20 min, and fixed again for 15 min with 10% tri-chloroacetic acid on ice. Fixed cells were permeabilized for 5 min with 0.1% Triton X-100 (PBS-T) in PBS and blocked for 1 h with 3% normal goat serum, 2% bovine serum albumin and 1% skim milk in PBS-T. Fixed/permeabilized cells were then stained with anti-Rab11 mAb (47, 1:100 dilution; BD Biosciences) diluted in Can Get Signal immunostain (TOYOBO, Osaka, Japan).

### Subcellular fractionation analyses

Subcellular fractionation analyses were performed as described previously [24]. Cell pellets were resuspended in 750 μl of ice-cold TEE buffer (10 mM Tris-HCl [pH 7.4], 1 mM EDTA and 1 mM EGTA) and then incubated for 1 min on ice to osmotically swell the cells. Thereafter, 250 μl of ice-cold homogenization buffer (1 M sucrose in TEE buffer) and 10 μl of 100x Complete Protease Inhibitors (Roche Diagnostics) were added, and the cell suspension was homogenized by 50 strokes using a 2-ml loose-fitting Dounce homogenizer on ice. The disrupted cells were transferred to 1.5-ml microcentrifuge tubes, and post-nuclear supernatant was obtained by centrifugation of the cell homogenate for 10 min at 1,000 × *g*, followed by centrifugation for 20 min at 8,000 × *g*. The resultant supernatant was equilibrated in 100 mM sodium carbonate [pH 11.0] for 5 min and sonicated with 3 × 5-s bursts in a UP 200 H sonicator (Dr. Hielscher GmbH, Teltow, Germany) at amplitude setting 50. Sonication rounds were intercalated by 1-min intervals to avoid heating the sample. The sonicated sample (2 ml) was transferred to a 12 ml polycarbonate ultracentrifuge tube and adjusted to 40% (w/v) sucrose by adding an equal volume of 80% (w/v) sucrose in TEE buffer to give a final volume of 4 ml. The sample was overlaid with 4 ml of 35% (w/v) sucrose, followed by 4 ml of 5% (w/v) sucrose, and subjected to discontinuous equilibrium sucrose density-gradient centrifugation for 4 h at 180,000 × *g* using a RPS40T swing rotor (Hitachi Koki, Tokyo, Japan). After centrifugation, 1 ml gradient fractions were harvested beginning from the top of the tube. These 12 1-ml fractions were dialyzed against PBS overnight at 4°C. Dialyzed fractions were concentrated using an Amicon Ultra Centrifugal Filter 3K (Merck Millipore).

### Internalization assay using cleavable sulfo-NHS-SS-biotin

Cell surface proteins on IMS32 cells were biotinylated using 1 mg/mL EZ-Link^™^ sulfo-NHS-SS-biotin (Thermo Fisher Scientific, Inc., Rockford, IL, USA) in HBSS (138 mM NaCl, 5.3 mM KCl, 1.3 mM CaCl_2_, 0.5 mM MgCl_2_, 0.4 mM MgSO_4_, 0.3 mM Na_2_HPO_4_, 0.4 mM KH_2_HPO_4_, 10 mM HEPES [pH 7.4], 5.5 mM Glucose) for 1 h at 4°C, after which excess biotin was quenched with HBSS containing 20 mM glycine. Thereafter, the cells were incubated at 37°C in DMEM supplemented with 5% FBS for various time periods. Remaining cell surface biotin was stripped twice using reducing buffer (50 mM GSH, 70 mM NaCl, 80 mM NaOH, 0.2% BSA) for 20 min at 4°C, then quenched with HBSS containing 50 mM iodoacetamide. Cells were then lysed in 0.2 mL RIPA buffer supplemented with Complete Protease Inhibitors (Roche Diagnostics). Biotinylated proteins (100 μg) were precipitated using Neutr-Avidin-agarose beads (Thermo Fisher Scientific, Inc.), eluted with SDS-PAGE sample buffer containing dithiothreitol, and used for western blot analysis.

### Biotinylation recycling assay using cleavable sulfo-NHS- SS-biotin

Cell surface proteins on IMS32 cells were biotinylated using 1 mg/mL EZ-Link^™^ sulfo-NHS-SS-biotin as described above, after which they were incubated in DMEM supplemented with 5% FBS for 15 min at 37°C to induce TfnR internalization. Remaining cell surface biotin was stripped twice with the reducing buffer for 20 min at 4°C, then quenched with HBSS containing 50 mM iodoacetamide. Cells were then subjected to a second round of incubation for 0–30 min at 37°C to allow internalized proteins to recycle back to the cell surface. Biotinylated proteins recycled to the cell surface were again stripped of biotin using the reducing buffer and quenched with HBSS containing 50 mM iodoacetamide. Cells were then lysed in 0.2 mL RIPA buffer with Complete Protease Inhibitors (Roche Diagnostics), and 100 μg of biotinylated proteins were precipitated using Neutr-Avidin-agarose beads (Thermo Fisher Scientific, Inc.). The precipitates were analyzed by western blotting.

### Fluorescent transferrin recycling assay

Hela cells were serum starved for 3 h in DMEM containing 0.2% BSA and then incubated with 5 ng/ml Alexa Fluor 594-conjugated Transferrin (Alexa-594-Tfn, Thermo Fisher Scientific, Inc.) for 20 min at 37°C. After first washing out the unbound Alexa-594-Tfn using an acid solution (0.2 M acetic acid, 0.5 M NaCl in PBS), cells were washed three times with PBS and incubated for various periods of time at 37°C in DMEM containing 25 mM HEPES [pH 7.4], 20 mM glucose, 1% BSA and 50 ng/ml of Holo-Tfn (Sigma–Aldrich). After washing with PBS, cells were fixed for 10 min with 4% PFA and processed for immunocytochemistry. The signal intensity of Alexa-594-Tfn per 20 μm^2^ in at least 20 cells was measured using FV10-ASW 3.0 software (Olympus) in 3 independent experiments.

## Results

### SIMPLE binds to PI4P through the SIMPLE-like domain

**Fig 1:**
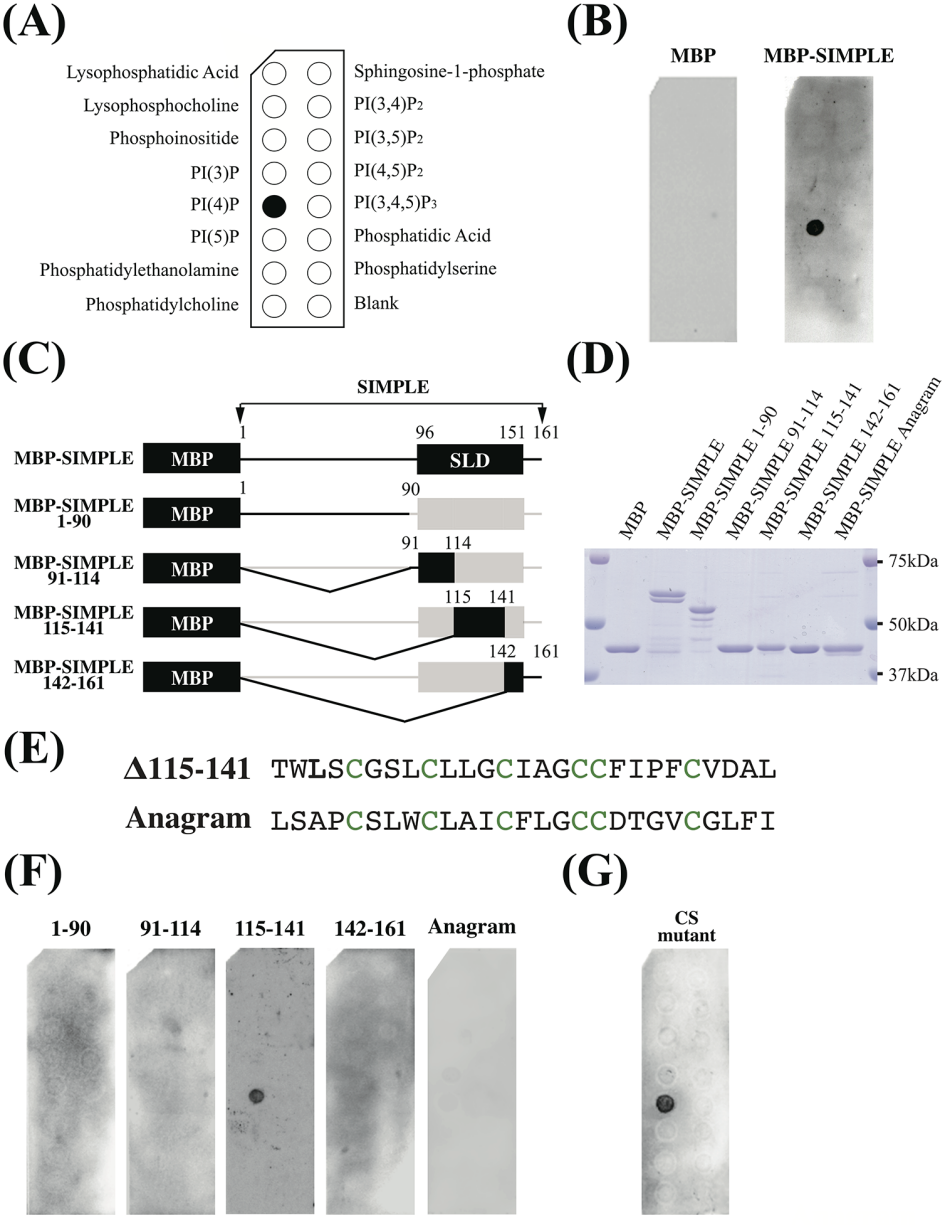
SIMPLE binds specifically to PI4P through the SIMPLE-like domain. (A) Schematic representation of the indicated membrane lipids on a PIP strip. (B) MBP-SIMPLE and MBP alone, as a negative control, were incubated with a PIP strip and detected as described in the Materials and Methods. (C) Schematic representation of SIMPLE deletion constructs. Numbers indicate SIMPLE amino acid residues. SLD is the SIMPLE-like domain. (D) CBB-stained bands of purified MBP or MBP-tagged human-SIMPLE. (E) Alignment of 115–141 and the 115–141 anagram. Conserved cysteine residues are indicated in green. (F) Binding of MBP-fusion proteins (see D) to PIP strips: each protein (5 nM) was added to a PIP strip. (G) Binding of MBP-SIMPLE with cysteine-to-serine (CS) mutantion to PIP strips.

We investigated the lipid-binding ability of maltose binding protein-fused SIMPLE (MBP-SIMPLE) using an overlay assay in which various lipid molecules were immobilized (Phosphatidyl inositol phosphate (PIP) Strip; Fig 1A). As shown in Fig 1B, MBP-SIMPLE clearly bound to PI4P, but not to any other lipids. No lipid binding was detected with MBP alone. Even when exposure time of the blot was increased, we could not detect binding of SIMPLE to any lipid besides PI4P (data not shown).

To determine the region responsible for binding PI4P, we constructed several truncated forms of MBP-SIMPLE (Fig 1C), which were purified on amylose resin (Fig 1D). In a PIP strip assay, MBP-SIMPLE (115–141) bound to PI4P (Fig 1F). To test whether PI4P-binding by MBP-SIMPLE (115–141) was specific, we tested an anagram of MBP-SIMPLE (115–141) in which the amino acid residues were shuffled randomly (Fig 1E). As shown in Fig 1F, this anagram did not bind to lipid molecules. These results indicate that SIMPLE (115–141) contains the region responsible for binding PI4P. Given that FYVE domains bind PI3P in a cysteine-dependent manner [23], we tested whether a conserved cysteine was analogously necessary for binding PI4P. Using a mutant in which serine was substituted for cysteine within SIMPLE (115–141) (CS mutant), we found that the CS mutant bound to PI4P as well as native MBP-SIMPLE (Fig 1G). Thus, SIMPLE appears to bind specifically to PI4P through the SLD but in a cysteine-independent manner.

### SIMPLE co-localizes with PI4P in cells

**Fig 2:**
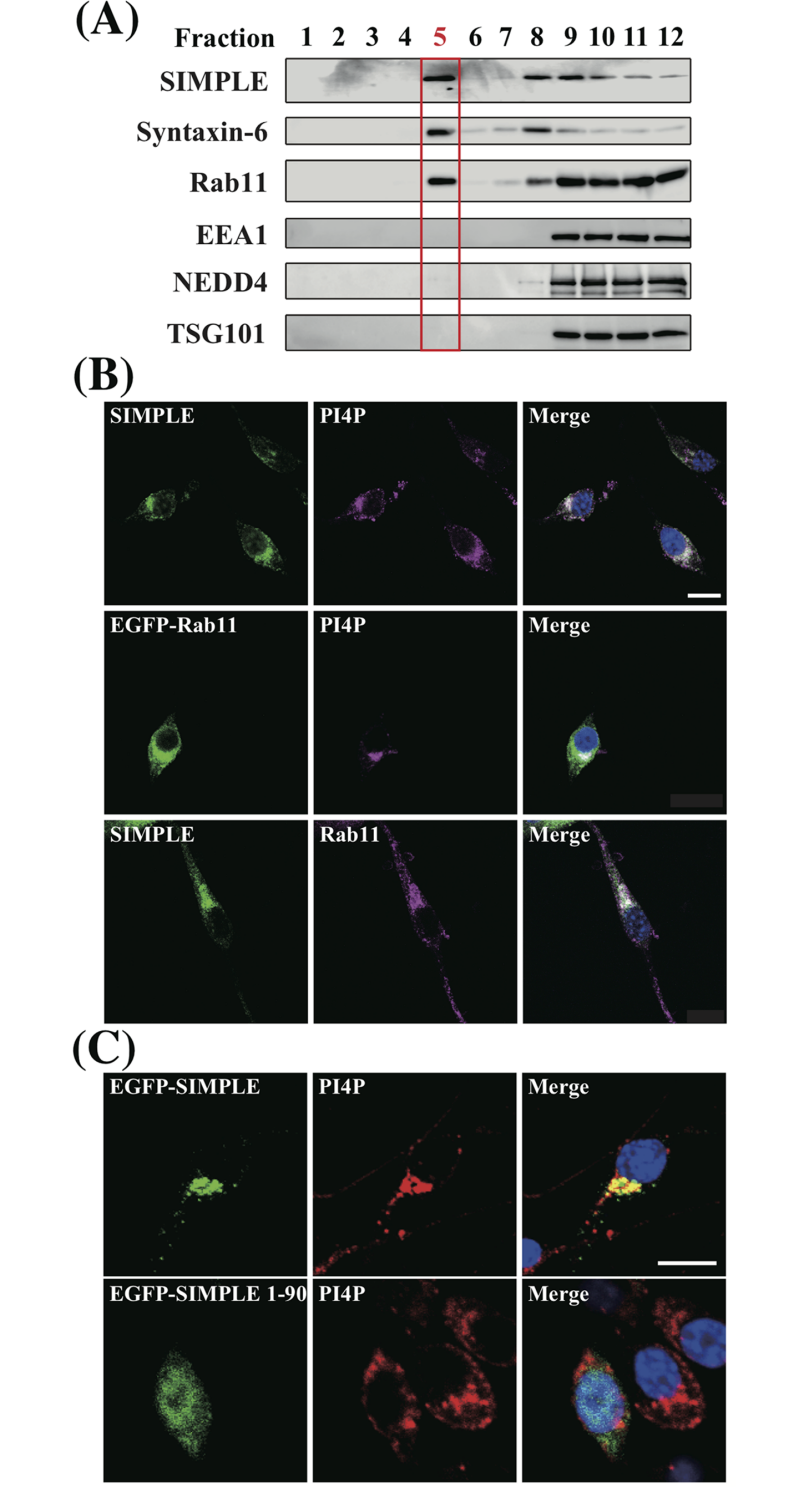
Localization of endogenous SIMPLE to organelles enriched in PI4P and recycling endosomes in IMS32 cells. (A) Subcellular fractionation of IMS32 cell lysates was conducted by discontinuous sucrose density-gradient centrifugation and collected into 12 fractions. Equal volumes from each fraction were subjected to SDS-PAGE followed by immunoblot analysis. Previous studies suggested fraction 5 (Red) was rich in PI4P [24]. (B) Co-localization of immunofluorescence within IMS32 cells imaged using confocal microscopy. Left-side panels show endogenous SIMPLE or transiently expressed EGFP-Rab11 (Green). Middle panels show endogenous Rab11 or PI4P in the same cells labeled with specific antibodies (Purple). Merged images are shown on the right with DAPI-stained nuclei (blue). Scale bar, 20 μm. (C) IMS32 cells were transiently transfected with EGFP-tagged full length SIMPLE or SIMPLE (1–90). Twenty-four hours post-transfection the cells were immunostained with anti-PI4P antibody. Left-side panels show EGFP-tagged full length SIMPLE or SIMMPLE (1–90) (Green). Middle panels show PI4P in the same cells labeled with specific antibodies (Red). Merged images are shown on the right with DAPI-stained nuclei (blue). Scale bar, 20 μm.

PI4P is the most abundant phosphoinositide in the membranes of the TGN and REs [7, 24]. Because SIMPLE binds PI4P, we investigated whether SIMPLE colocalizes in organelles enriched in PI4P. First, discontinuous sucrose density-gradient fractionation, which specifically enriches proteins from the TGN and REs, was employed to reveal the localization of SIMPLE in the IMS32 mouse-derived Schwann cell line [24]. As shown in Fig 2A, a portion of SIMPLE co-fractionated with syntaxin-6 (a TGN-resident protein) and Rab11 (a RE-resident, PI4P-binding protein) in fraction 5. This fraction (#5), contained TGN and RE, but did not include NEDD4 or TSG101, which also reportedly interact with SIMPLE [14, 15, 17, 18]. In contrast with previous reports that SIMPLE localizes in EEs [14, 15], SIMPLE in this fraction (#5) did not co-migrate with EEA1 (an EE-resident protein) in IMS32 cells. These results suggest that, in IMS32 cells, SIMPLE localized in part to the TGN and REs, and not only to EEs.

To further investigate the localization of SIMPLE within organelles enriched in PI4P, fixed and permeabilized IMS32 cells were immunohistochemically analyzed using anti-SIMPLE and anti-PI4P antibodies. The distribution of SIMPLE mostly overlapped that of PI4P in the perinuclear region (Fig 2B, upper panels). In EGFP-tagged Rab11-expressing cells, the PI4P signal overlapped that of EGFP-Rab11 (Fig 2B, middle panels), and SIMPLE also mostly colocalized with Rab11 in the perinuclear region (Fig 2B, lower panels). These results suggest that SIMPLE localizes with Rab11 within organelles enriched in PI4P, especially REs.

To address the importance of the SLD for localization of SIMPLE in organelles enriched in PI4P, EGFP-tagged SIMPLE and EGFP-tagged SIMPLE (1–90) were transfected into IMS32 cells, and immunostained with anti-PI4P antibody. Although the full-length SIMPLE colocalized with PI4P in the perinuclear region, SIMPLE (1–90), which lacks SLD completely, lost its colocalization with PI4P and distributed throughout the cytoplasm and nucleus (Fig 2C). These results further support the necessity of the SIMPLE SLD for its localization in organelles enriched in PI4P.

### SIMPLE regulates exocytosis of recycled vesicles

**Fig 3:**
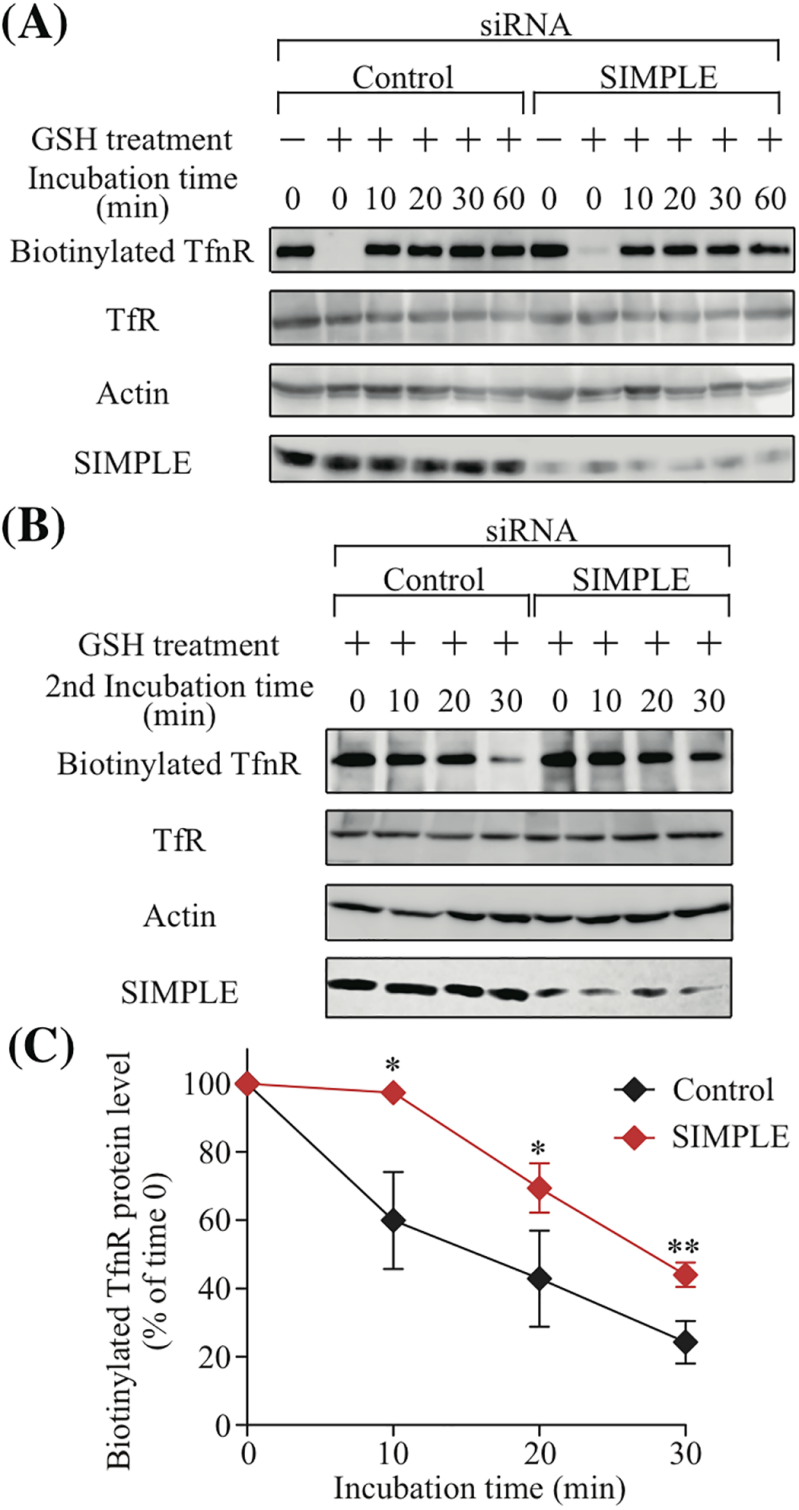
SIMPLE knockdown affects recycling of TfnR, but not its internalization. (A) IMS32 cells transfected with control or SIMPLE siRNA were treated with sulfo-NHS-SS-biotin at 4°C, after which the cells were incubated at 37°C for the indicated amount of time. Cells were then treated with reducing buffer containing GSH to remove remaining biotin from the plasma membrane, and cell lysates were precipitated with neutravidin-agarose beads. Precipitates were analyzed using Western blot with an anti-TfnR mAb (biotinylated TfnR), and cell lysates used for precipitation (10% input) were probed with anti-TfnR mAb (TfnR), anti-β-actin mAb and anti-SIMPLE pAb. (B) After cell-surface biotinylation using sulfo-NHS-SS-biotin, IMS32 cells transfected with control or SIMPLE siRNA were incubated for 15 min. Remaining surface biotin was stripped using reducing buffer, and cells were incubated for the indicated periods of time. Biotinylated proteins that recycled back to the cell surface were again treated with reducing buffer, and cell lysates were precipitated with neutravidin-agarose beads. Precipitates were analyzed by Western blot with an anti-TfnR antibody, and cell lysates (10% input) were probed with the indicated antibodies. (C) The amount of biotinylated TfnR in (B) was quantified using Image J software. The results shown are means ± S.D. of the ratio between biotinylated TfnR at each time point and biotinylated TfnR at time zero from three independent experiments. Values at time zero are set to 100%. P-values (control cells vs. SIMPLE knockdown cells at 10, 20, 30 min) are determined using a t-test. *P<0.05, **P<0.001.

Rab11 is known to be involved in regulating exocytosis of recycled vesicles [25]. Because SIMPLE co-localized with Rab11, we assessed the possible contribution of SIMPLE to protein sorting in REs by monitoring Transferrin Receptor (TfnR), one of the most extensively studied recycled proteins [26, 27]. We evaluated the effect of a SIMPLE knockdown on the internalization of TfnR as follows. Cell surface proteins were labeled with a cleavable biotinylation reagent (sulfo-NHS-SS-biotin), after which the treated cells were incubated for various amounts of time to allow internalization of the biotinylated proteins. Any biotin still attached to the cell surface was eliminated using glutathione (GSH), a reducing agent. As shown in Fig 3A, when the cells were incubated for 10–30 min, biotin-TfnRs in cells transfected with SIMPLE siRNA were internalized as well as in cells transfected with control siRNA. Apparently, SIMPLE does not affect TfnR internalization. On the other hand, the biotin-TfnR signal in the control siRNA-treated cells was decreased by elimination from the surface after 30 min of re-culture following the first reducing treatment, while the signal in SIMPLE siRNA-treated cells remained (Fig 3B). After assigning a value of 100% to the biotin-TfnR signal intensity at time zero in the control and SIMPLE knockdown cells, we plotted the relative biotin-TfnR signal intensities at the indicated times (Fig 3C). Although the biotin-TfnR signal decreased over time in both treatment groups, this decline was delayed in SIMPLE knockdown cells (Fig 3C). These observations suggest SIMPLE contributes to regulating exocytosis of recycled vesicles.

**Fig 4:**
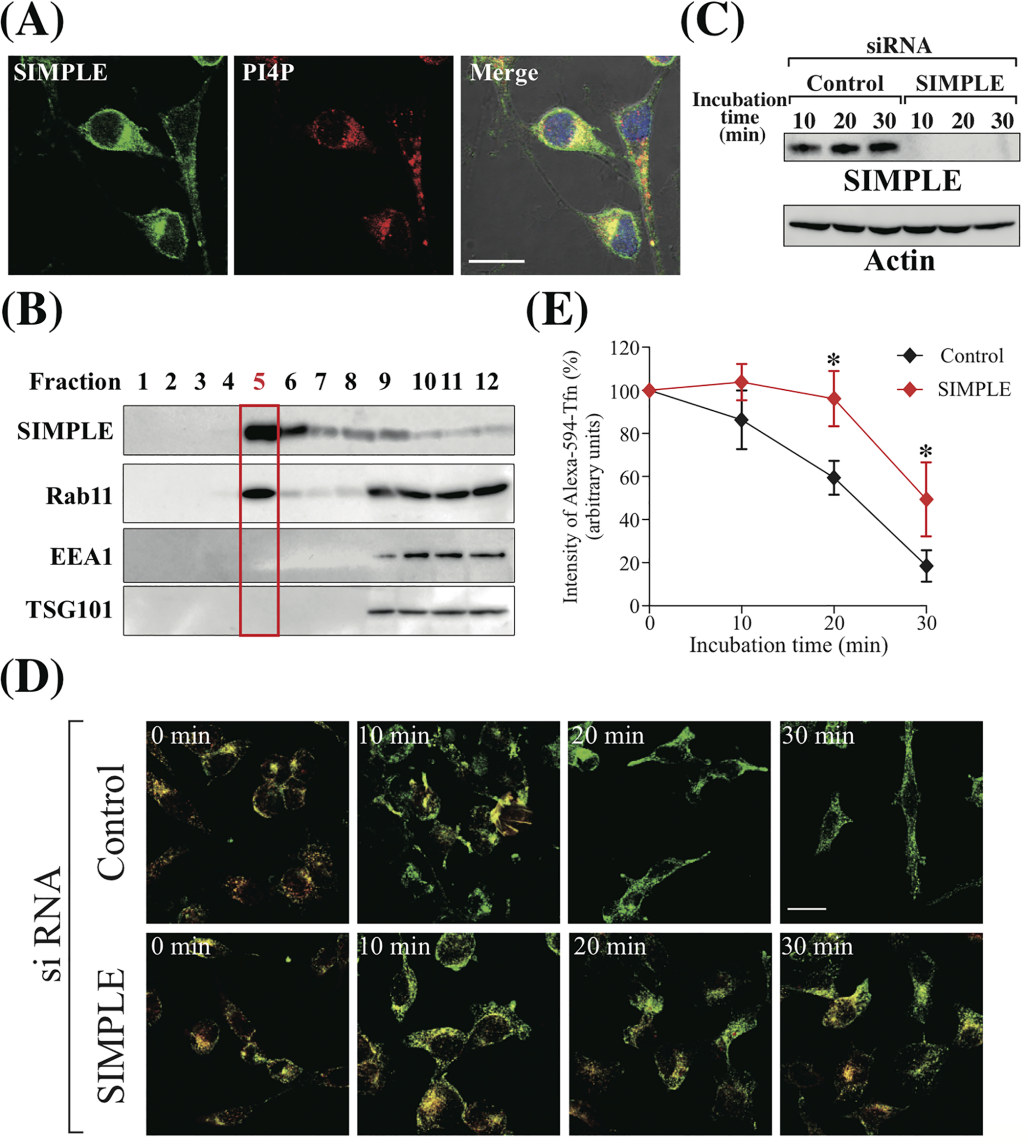
SIMPLE knockdown impedes Tfn recycling in Hela cells. (A) Co-localization of immunofluorescence within Hela cells imaged using confocal microscopy. Left-side panels show endogenous SIMPLE (Green). Middle panels show PI4P in the same cells labeled with specific antibodies (Red). Merged images are shown on the right panels with DAPI-stained nuclei (blue). Scale bar, 20 μm. (B) Subcellular fractionation of Hela cell lysates separated into 12 fractions through discontinuous sucrose density-gradient centrifugation. Equal volumes of each fraction were subjected to SDS-PAGE followed by immunoblot analysis. Previous studies suggested fraction 5 (Red) is enriched in PI4P [24]. (C) Hela cells were transfected with control or SIMPLE siRNA and cultured for 48 h. After serum starvation, Alexa-594-Tfn treatment and incubation for the indicated periods of time, cell lysates were prepared and subjected to SDS-PAGE (30 μg of total protein/lane). Immunoblots were probed with anti-SIMPLE mAb or anti-β-actin mAb. (D) Hela cells transfected with control or SIMPLE siRNA were serum starved for 3 h and then incubated with Alexa-594-Tfn at 37°C for 1 h. After washing out unbound Alexa-594-Tfn, cells were incubated at 37°C for the indicated amount of time and processed for immunostaining with anti-TfnR mAb. Fluorescent images of Alexa-594-Tfn (red) and TfnR (green) are shown along with merged images. Scale bar, 20 μm. (E) The intensity of Alexa-594-Tfn signal in Hela cells was expressed as signal intensity per unit area. At each time point, the signal intensity of at least 20 cells was measured in three independent experiments. The results shown are means ± S.D. of Alexa-594-Tfn signal at each time normalized to signal at time zero, which are assigned a value of 100% (n = 3). P-values (control cells vs. SIMPLE knockdown cells at 10, 20, 30 min) are determined using a t-test. *P<0.05.

As SIMPLE is also expressed in various tissues other than Schwann cells [8, 14], we also examined the effect of SIMPLE on Transferrin (Tfn) recycling in Hela cells. First, to confirm the localization of SIMPLE and PI4P, Hela cells were immunostained with anti-SIMPLE and anti-PI4P antibodies. As in IMS32 cells, the distribution of SIMPLE overlapped with that of PI4P in the perinuclear region in Hela cells (Fig 4A). Moreover, subcellular fractionation showed that SIMPLE co-fractionated with Rab11 (Fig 4B).

To assess the effect of a SIMPLE knockdown on Tfn recycling, Hela cells transfected with control or SIMPLE siRNA were incubated with fluorescently labeled transferrin (Alexa-594-Tfn) for 20 min at 37°C. After washing out unbound Alexa-594-Tfn, cells were incubated at 37°C for various amounts of time to monitor the recycling of Alexa-594-Tfn, and processed for immunostaining with anti-TfnR antibody. SIMPLE siRNA effectively suppressed endogenous SIMPLE expression at the indicated incubation times (Fig 4C). In addition, it can be seen in Fig 4D that yellow signal, which corresponds to merged Alexa-594-Tfn (red fluorescence) and TfnR (green fluorescence) signal, was present in the control siRNA-treated cells but was completely absent after 20 min of re-culture. By contrast, in SIMPLE siRNA-treated cells, the merged signal persisted after 30 min. To evaluate the difference between control and SIMPLE knockdown cells, Alexa-594-Tfn signal intensity at time zero in control and SIMPLE knockdown cells was individually set to 100%, and the relative Alexa-594-Tfn signal intensities at the indicated times were plotted (Fig 4E). Although the Alexa-594-Tfn signal decreased over time in both treatment groups, the decline in Alexa-594-Tfn signal was delayed in SIMPLE knockdown cells (Fig 4E). These observations further suggest that SIMPLE regulates exocytosis of recycled vesicles.

## Discussion

In this study, we demonstrated that SIMPLE binds specifically to PI4P through the 115–141 region within the SLD, and that this binding was cysteine-independent. Recently, two groups independently revealed that SIMPLE is a monotopic zinc-binding protein, and that the function of cysteine residues outside the 114–139 hydrophobic helical region is to mediate zinc binding for stabilization of the protein structure [12, 13]. On the other hand, the FYVE domain, which binds PI3P in a cysteine-dependent manner, requires two zinc ions for proper structure [23]. Our finding that the CS mutant of SIMPLE also binds PI4P indicates that the 115–141 region of the SLD represents a novel PI4P binding domain that differs from the FYVE domain.

Ho *et al*. also reported that the 114–139 hydrophobic helical region interacts with phosphatidylethanolamine (PE) [12]. Using liposome sedimentation assays, we tested the ability of SIMPLE to bind PI4P. However, we were unable to analyze PI4P binding activity of SIMPLE in this assay because SIMPLE has a hydrophobic region and easily integrates into liposomes constructed with PE and phosphatidylcholine (PC) (data not shown). Our PIP-strip assay did not reveal binding of SIMPLE to PE or PC (Fig 1B and 1F). Further investigation is needed to determine whether SIMPLE can bind PE.

Previous studies have shown that SIMPLE interacts with WWOX, Itch, Hrs and STAM1, as well as NEDD4 and TSG101 [12, 15, 17, 18, 20]. Although these proteins reportedly relate to ESCRT and regulate trafficking of ubiquitinated proteins to MVBs [28, 29], Hrs also appears to regulate recycling back to the plasma membrane of calcitonin receptor-like receptor [30]. Because our subcellular fractionation assays, which revealed that SIMPLE co-fractionated with syntaxin-6 and Rab11 (fraction 5), did not include NEDD4 or TSG101, another SIMPLE binding protein, possibly Hrs, may contribute to the recycling function of SIMPLE. Further investigation is warranted to clarify how SIMPLE contributes to recycling in the TGN and/or REs.

Several mutations in the *SIMPLE* gene have been identified as a cause for one of the subtypes of Charcot-Marie-Tooth disease (CMT) [31, 32]. These mutations damage myelin sheaths composed of several myelin-related proteins, including PMP22 and peripheral myelin protein zero (P0). Because these proteins were produced by Schwann cells, we investigated SIMPLE localization and function using the IMS32 mouse-derived Schwann cell line [33]. Additionally, since SIMPLE is expressed in several tissues other than Schwann cells, we also examined SIMPLE localization and function in Hela cells. We found that the localization of SIMPLE overlapped that of PI4P and Rab11, two RE markers, in the perinuclear region of both IMS32 and Hela cells (Figs 2B and 4A). Our subcellular fractionation assay revealed that SIMPLE co-fractionated with syntaxin-6 (a TGN-resident protein) and Rab11. Furthermore, we observed that SIMPLE knockdown affected Tfn and TfnR recycling in both IMS32 and Hela cells (Figs 3B and 3C, 4D and 4F). Although SIMPLE has been reported to contribute to endosomal sorting and/or exosome production [15, 16], our results suggest that some SIMPLE within cells localizes in the TGN and/or REs, and contributes to recycling not only in Schwann cells but also in other cell types.

It was recently reported that SH3TC2 and NDRG1, which lead to CMT4C and CMT4D upon mutation, are localized in REs and are involved in regulating membrane protein recycling [34–36]. In addition, aberrant myelination was observed when a dominant negative Rab11 mutant was expressed in Schwann cells [35]. Moreover, REs are known to be involved in PMP22 trafficking, which is one of the components of compact peripheral nerve myelin [37]. These observations, coupled with our findings that SIMPLE contributes to protein recycling, suggest that SIMPLE may affect peripheral nerve myelination by regulating the recycling of membrane proteins, such as PMP22.

In summary, our results indicate that SIMPLE localizes to the TGN and/or REs through binding to PI4P by the SLD, through which it regulates protein trafficking. The SLD is a novel type of PI4P binding domain, and other SLD containing proteins may localize at the TGN and/or REs and might regulate protein trafficking.
